# Seroprevalence and Potential Risk Factors of Toxocariasis among General Population in Southwest Iran: Implications on the One Health Approach

**DOI:** 10.1155/2024/4246781

**Published:** 2024-02-13

**Authors:** Masoud Foroutan, Aida Vafae Eslahi, Shahrzad Soltani, Naser Kamyari, Ehsan Moradi-Joo, Jean-Francois Magnaval, Milad Badri

**Affiliations:** ^1^Research Center for Environmental Contaminants (RCEC), Abadan University of Medical Sciences, Abadan, Iran; ^2^Department of Basic Medical Sciences, Faculty of Medicine, Abadan University of Medical Sciences, Abadan, Iran; ^3^Medical Microbiology Research Center, Qazvin University of Medical Sciences, Qazvin, Iran; ^4^Department of Public Health, School of Health, Abadan University of Medical Sciences, Abadan, Iran; ^5^Department of Management and Medical Information Sciences, Kerman University of Medical Sciences, Kerman, Iran; ^6^Department of Medical Parasitology, Faculty of Medicine, Paul Sabatier University, 37 Allees Jules-Guesde, 31000 Toulouse, France

## Abstract

Toxocariasis is one of the most common zoonotic diseases distributed worldwide. This study aimed to estimate the seroprevalence of anti-*Toxocara* immunoglobulin G (IgG) antibodies and the associated risk factors among general populations living in urban and rural areas of Abadan and Khorramshahr cities in Khuzestan Province, Southwest Iran. This cross-sectional study was conducted between March and September 2022. There were 363 participants (190 females and 173 males) aged from <20 to ≥60 years old. Anti-*Toxocara* IgG antibodies in serum samples were measured using a commercially available enzyme-linked immunosorbent assay (ELISA). A structured questionnaire was employed to collect information regarding sociodemographic status and probable risk factors associated with toxocariasis. It was found that the seroprevalence rate in males (15.0%, 95% CI = 10.47–21.11) was higher than in females (10.5%, 95% CI = 6.92–15.70). Moreover, we observed that the seroprevalence was higher in participants at younger ages compared to other age ranges (COR = 2.55, 95% CI = 0.92–7.12, *p* =0.073). The findings of the univariate analysis revealed that residency in rural areas (*p*  < 0.001), using unpurified water (*p*  < 0.001), contact with dog (*p* =0.002), contact with soil (*p*  < 0.001), consumption of improperly washed vegetables (*p*  < 0.001), and history of drinking untreated water (*p*  < 0.001) were risk factors associated with toxocariasis. Further comprehensive studies with a focus on humans and animals should be designed in different areas of the Province. The data represented by the current study are useful to health policymakers to consider precise surveillance and effective prevention measures to control this zoonotic infection among general populations.

## 1. Introduction

Soil-transmitted helminths (STHs) have been included to the list of 17 neglected tropical diseases (NTDs) reported by the World Health Organization (WHO) due to the consequences such as considerable rate of morbidity, severe poverty, and disability-adjusted life years (DALYs) lost [1]. According to formal reports, there are over 1.5 billion persons infected with STHs globally, with tropical nations having the highest prevalence [2, 3]. More than 135,000 deaths are thought to occur annually as a result of STHs [4]. Toxocariasis is a neglected infectious disease of zoonotic importance caused by the globally distributed parasites of the genus *Toxocara* [5, 6]. It is among the top five neglected parasitic diseases of public health concern [6, 7]. Based on available data, ∼2 billion of the world population are susceptible to acquire the infection [8]. The causal agent of the infection is *Toxocara canis* (the dog roundworm), and to a minor extent, *T. cati* (the cat roundworm), both of which inhabit the lumen of definitive hosts [7]. Dogs are the canids that thoroughly have adapted to human habitation, and they improve the physical, mental, and social health of their owners, particularly children [9]. On the other hand, domestic dogs and cats are the key reservoirs of the infection, and they have a major part in the epidemiology of human toxocariasis, as they frequently excrete into human environment [10–12]. The global estimations revealed that the prevalence of *Toxocara* in dogs was 11.1% with ≥100 million dogs infected with this parasite globally [12].

Humans as the accidental host acquire the infection through ingestion of water, and food, or contact with soil contaminated with the infective (embryonated) eggs of the parasites from dog and cat feces [13–15]. The meat of paratenic hosts (e.g., chicken and cow livers) if consumed raw or uncooked can be a source of infection [14, 15]. It also must be noted that the presence of the eggs on the hair of domestic pets, mostly dogs, can be an evidence for possible direct transmission of these agents via close exposure of the owners with their pets [16].

Human toxocariasis (HT) is typically asymptomatic, except the conditions such as visceral larva migrans (VLM), ocular toxocariasis (OT), and neurological toxocariasis (NT), which occur as a result of larval migration [17]. The manifestations range from a nonspecific picture, including abdominal pain, intermittent diarrhea, frequent headache, otorhinolaryngeal allergy, paresthesia, arthralgia and/or myalgia, frequently chronic weakness, chronic cough, cutaneous allergy, pruritus *sine materia*, facial and/or hand edema, conjunctivitis, wheeze that appears in covert toxocariasis (CT), to fever, eosinophilia, and hepatomegaly in visceral toxocariasis; meningitis, myelitis, encephalitis, seizures, and cerebral vasculitis in neurotoxocariasis; and vision problems and retinal scarring in ocular toxocariasis [18–20].

*Toxocara* spp. eggs are not infective on shedding and they develop to infective stage under favorable environmental conditions and during a period of time [21]. Based on a review paper with meta-analysis approach, the seroprevalence rate of HT was found to be 19.0% (95% CI = 16.6%–21.4%) globally, demonstrating that almost 20% of the human population has been exposed to infection caused by *Toxocara* parasites [22]. The current strategies to limit risk of transmission from pets to humans are concentrated on elimination of mature parasites in animals and removal of excretes and covering sand pits in areas such as parks and playgrounds that are used for recreational activities [23].

Seroprevalence studies on toxocariasis play a key role in understanding the prevalence and risk factors associated with this parasitic infection in humans. The information obtained from these studies contributes to public health planning, disease monitoring, and the development of targeted interventions to reduce the impact of toxocariasis on both human and animal populations. Moreover, these studies support a One Health approach by considering the interactions between humans, animals, and the environment. This integrated approach is essential for addressing complex issues related to zoonotic diseases. The overall seroprevalence of toxocariasis in Iran was found to be 9.3%, ranging from 0.84% to 29% [24]. The majority of these studies in Iran were conducted in school children, since it is usually regarded as a pediatric disease [25]. Considering the public health concern associated with toxocariasis, and due to the limited surveys, the present study aimed to estimate the seroprevalence and related risk factors of the infection in general populations living in urban and rural areas of Abadan and Khorramshahr cities (Khuzestan Province, Southwest Iran).

## 2. Materials and Methods

### 2.1. Study Area

Abadan city (30.3666° N, 48.2755° E) is situated in the Khuzestan province, southwest of Iran. It is bounded in the East by the Bahmanshir outlet of the Karun River and in the West by the Arvand waterway, close to the border of Iran and Iraq. Its current population is almost 350,000 people. Summers are dry and intensely hot, with temperature up to 53°C. The temperature ranges from 53°C in summer to −4°C in winter. The city has a mean temperature of 25.5°C, an average annual precipitation of 153.3 mm, and an average humidity of 45% (https://en.wikipedia.org/wiki/Abadan,_Iran) [26, 27]. Khorramshahr city (30.4256° N latitudes, 48.1891° E longitudes) is located nearly 10 km north of Abadan in Khuzestan province. It is regarded as one of the most important ports in Iran and is situated at the confluence of the Karun and Arvand rivers. The population of the city (recorded in the 2006 census) is estimated at 155,224 people (https://en.wikipedia.org/wiki/Khorramshahr_County) [28]. During the summer, the weather becomes notably hot, while in winter, it tends to be mild. The average maximum temperature peaks at 47.5°C, and the minimum temperature averages around 9°C. Additionally, there is an average annual precipitation of 150.6 mm [29].

### 2.2. Study Population and Sampling Strategy

The current cross-sectional study was carried out between March and September 2022 and it was approved by the Ethics Committee of Abadan University of Medical Sciences (Approval No: IR.ABADANUMS.REC.1400.103). Some inclusion criteria were defined in this research project as follows: (1) participants ≥15 years old; (2) residency in urban and rural regions of Khorramshahr or Abadan cities; and (3) volunteer to participate in the study upon obtaining a written informed consent. It should be noted that unwilling to take the blood sample and generally not feeling well in the time of sampling were the exclusion criteria. A structured questionnaire was attached to the consent form to collect information regarding the sociodemographic features (age, gender, educational level, and residential status) of the participants (Tables 1 and 2). A total of 363 individuals aged from <20 to ≥60 years old participated in this study; 190 (52.3%) were female and 173 were male (47.7%). The majority of the participants were 20–39 years of age (43.3%). To maintain a sufficient sample size for analysis, the participants were divided into four age groups as follows: <20, 20–39, 40–59, and ≥60 years old.

Regarding the level of education, most of the participants (71.6%) were diploma or lower and 103 (28.4%) had a university degree.  Table 1 represents the details of the sociodemographic characteristics.

The other criteria including history of contact with dog, contact with soil, source of drinking water, history of drinking untreated water, consumption of improperly washed vegetables, and awareness regarding *Toxocara* infection and its transmission routes were considered as risk factors (Table 1). Additionally, some clinical symptoms associated with toxocariasis such as history of asthma, skin allergic disorders, ophthalmic disorders or blurred vision, and epilepsy were included in the questionnaire (Table 2).

### 2.3. Blood Sampling and Detection of Anti-*Toxocara* IgG Antibodies

Three hundred and sixty-three blood samples were collected, from both Abadan and Khorramshahr cities. About 5 ml of blood was collected by venipuncture by paramedical staffs. Then, the blood samples were centrifuged at 1,700× *g* for 5′. The obtained sera stored at −20°C before serological analysis. To detect anti-*Toxocara* immunoglobulin G (IgG) antibodies in serum, a commercial enzyme-linked immunosorbent assay (ELISA) kit from IBL (Hamburg, Germany; Catalog no. RE587) was used and the procedures were conducted according to the manufacturer's description.

### 2.4. Statistical Analysis

A database was developed using SPSS Statistics software, version 21 (SPSS Inc., Chicago, IL, USA). The seroprevalence rate was defined as the relative frequency with an exact binomial 95% confidence interval (CI). The Pearson's *χ*^2^ test was used to analyze differences between categorical variables and odds ratios (ORs) with 95% confidence intervals. The odds ratios (ORs) with 95% CIs were also estimated. The association between toxocariasis seropositivity (dependent variable) and risk factor (independent variables) was first tested by univariate logistic regression analysis. Additionally, a multivariate logistic analysis for significant risk factors identified in the univariate analysis was conducted, and ORs and 95% CIs were estimated to associate the potential variables. A *p* value less than 0.05 was regarded as statistically significant.

## 3. Results

### 3.1. Seroprevalence Rate of Anti-*Toxocara* IgG Antibodies

Anti-*Toxocara* IgG antibodies were detected in 46 (12.7%) of the 363 sera (Table 1). The seroprevalence rate in males (15.0%, 95% CI = 10.47–21.11) was higher than in females (10.5%, 95% CI = 6.92–15.70). Moreover, although not statistically significant, the probability of finding a seropositive male individual was 1.49 times higher than a seropositive female (COR = 1.49, 95% CI = 0.81–2.78, *p*=0.20; Table 1).

The age-based analysis found the highest seroprevalence in individuals <20 years of age with the rate at 28.9% (95% CI = 17.73–43.37) and the lowest in 20–39 years old with the rate at 7.0% (95% CI = 3.96–12.11; Table 1).

According to the residential area, the seroprevalence of toxocariasis in individuals living in rural areas (33.8%, 95% CI = 23.88–45.38) was significantly higher than those living in urban areas (7.5%, 95% CI = 5.03–11.14; *p* < 0.001) (Table 1).

Seroprevalence rates based on educational status revealed that participants with diploma/or lower contributed to most of the seropositive cases (14.2%, 95% CI = 10.50–19.0), in comparison to participants with university degree (8.7%, 95% CI = 4.66–15.78), although the differences were not statistically significant (*p*=0.156; Table 1).

The evaluations based on the other risk factors associated with toxocariasis infection showed that there was a significantly higher seroprevalence in participants who had the following histories: contact with dog (17.9%, 95% CI = 13.62–23.06), contact with soil (29.1%, 95% CI = 21.94–37.56), eating improperly washed vegetables (24.2%, 95% CI = 17.99–31.63), drinking untreated water (23.7%, 95% CI = 17.72–30.97), and consumption of unpurified drinking water (35.2%, 95% CI = 23.82–48.51; Table 1).

Concerning the clinical manifestations, it was found that toxocariasis was significantly associated with skin allergic disorders (OR = 6.25, 95% CI = 3.33–12.50, *p* < 0.001), asthma (OR = 5.26, 95% CI = 2.86–10.0, *p* < 0.001), and ophthalmic disorders or blurred vision (OR = 16.67, 95% CI = 9.09–33.33, *p* < 0.001). Moreover, although not statistically significant, the IgG seroprevalence of toxocariasis showed a trend toward higher rates in participants with epilepsy (OR = 1.79, 95% CI = 0.48–6.67, *p*=0.390; Table 2).

### 3.2. Potential Risk Factors for Toxocariasis

Univariate analysis identified the following risk factors for toxocariasis infection: residency in rural areas (COR = 6.27, 95% CI = 3.25–12.08, *p* < 0.001), using unpurified water (COR = 5.67, 95% CI = 2.86–11.23, *p* < 0.001), contact with dog (COR = 23.91, 95% CI = 3.23–100.0, *p*=0.002), contact with soil (COR = 10.0, 95% CI = 4.76–25.0, *p* < 0.001), consumption of improperly washed vegetables (COR = 6.67, 95% CI = 3.12–14.29, *p* < 0.001), and history of drinking untreated water (COR = 6.67, 95% CI = 3.23–14.29, *p* < 0.001; Table 1).

## 4. Discussion

In our study, we found that the rate of IgG antibodies against *Toxocara* in general population of Abadan and Khorramshahr cities was 12.7%. This was higher than the findings reported by recent studies in Fars (5.8%) [24], Khorasan (8%) [30], and Tehran (11.7%) [31], although our estimates were lower than the seroprevalence identified in Mazandaran (23.5%) [32].

Similar results were found by studies from other parts of the world: Estonia (12.0%), China (12.25%), Turkey (12.95%), Poland (13.0%), Greece (16%), and Austria (16.8%) [33–38].

The analysis of the data from our study showed that the IgG seroprevalence of *Toxocara* was higher in participants at younger ages compared to other age ranges (COR = 2.55, 95% CI = 0.92–7.12, *p*=0.073). Given the reasons such as playing with soil, geophagic behavior, and close contact with dogs and cats, the risk of exposure to toxocariasis is more significant at the first few years of life, and global studies confirmed that the children aged ≤10 are more susceptible to toxocariasis infection than older ages [39]. It has been evidenced that *Toxocara* seropositivity in children has a significant association with the history of geophagia [40].

Moreover, we found that the seroprevalence was higher in residents of the rural areas compared to those live in urban areas (COR = 6.27, 95% CI = 3.25–12.08, *p* < 0.001). Similarly, studies conducted by EbrahimiFard et al. and Shokouhi et al. in Iran revealed a higher prevalence of toxocariasis in cases living in rural areas [41, 42].

This may be related to the poor hygiene habits as well as abundance of stray and domesticated dogs in rural regions lead to enhanced level of environmental contamination with *Toxocara* eggs [22, 43]. A systematic review study has shown that the pooled prevalence of *Toxocara* infection in dogs was 17% in Iran [44].

Toxocariasis is common in less-industrialized countries and in socioeconomically vulnerable populations of industrialized countries [20]. In the present study, we observed that the seroprevalence was higher in individuals with lower educational level (diploma or lower) compared to university degree (COR = 1.72, 95% CI = 0.81–3.73, *p*=0.160). Populations with lower education status are more suffering with lower socioeconomic conditions that are related with occupational contact with soil or living in areas where high levels of environmental contamination with *Toxocara* spp. eggs are present [45].

The expanding distribution of *Toxocara* nematodes in different geographical regions is possibly due to global warming and increased mobility of humans and animals [8]. A review study revealed that the worldwide seroprevalence of toxocariasis had a growing trend in recent decades. The explanation for this can be the increasing tendency of people in having close contacts with the definitive hosts that often are pets. Moreover, the significant rate of global urbanization in recent years has led to an increasing number of pets and stray dogs or cats in many areas of the world [22]. The open excretion practice and the frequent presence of these animals in public places (e.g., parks, children's playgrounds, and beaches) may result in shedding of eggs into the environment. The eggs in the feces can become infective, and they can reach various water sources such as rivers, streams, sewage systems, and consequently, they enter agricultural fields, and contaminate vegetables [22, 46]. It is notable that population in Khuzestan are faced with inadequate allocation of health care facilities and environmental challenges such as dust storms, floods, water crises, and infrastructural deficiencies [47–50].

In accordance with these declarations, we have observed that contact with dog, contact with soil, eating improperly washed vegetables, drinking untreated water, and consumption of unpurified drinking water are possible risk factors for seropositivity. Parallel to our results, previous studies in Iran found a relationship between *Toxocara* seropositivity and history of contact with dogs [32, 51].

In this study, we observed a significant association between toxocariasis and skin allergy manifestations, and asthma. Comparatively, a prior report has shown an increased risk for allergic skin disorders in patients with *Toxocara* seropositivity (OR = 1.75, 95% CI = 1.16–2.64) [52]. In addition, a meta-analysis showed that there was a significantly higher prevalence of toxocariasis in asthma patients than in controls (OR = 3.36, 95% CI = 1.76–6.42) [53].

The observations regarding clinical symptoms we described in the current study also revealed a significant association between toxocariasis and ophthalmic disorders, which is in agreement with the report on global incidence of ocular toxocariasis ranged between 17 and 52 per 100,000 persons occuring annually [54].

## 5. Conclusion

This study provides valuable insights into the seroprevalence and potential risk factors associated with toxocariasis in the studied population. The overall seroprevalence of anti-*Toxocara* IgG antibodies in Abadan and Khorramshahr cities indicated a notable presence of *Toxocara* exposure in the community. The analysis of demographic factors revealed variations in seroprevalence rates among different groups. The age-based analysis demonstrated that individuals under 20 years of age presented the highest seroprevalence, emphasizing the vulnerability of younger age groups to toxocariasis. Additionally, residents in rural areas showed a significantly higher seroprevalence compared to their urban counterparts, suggesting a potential environmental influence on *Toxocara* exposure.

Risk factors such as contact with dogs, contact with soil, consumption of improperly washed vegetables, drinking untreated water, and using unpurified water were significantly associated with the infection, underscoring the importance of environmental and behavioral factors in the transmission of *Toxocara*. The findings underscore the importance of public health interventions aimed at reducing environmental exposure to *Toxocara* and raising awareness about preventive measures, especially in high-risk populations such as individuals residing in rural areas and those with specific behavioral practices.

Specific screening and treatment strategies, besides improving standards of sanitation and control of infection in dogs, are required for the control and prevention of the disease in the human population in these regions. Exclusive studies with a focus on humans, dogs, and environmental health should be carried out in different areas of Khuzestan Province. The outcome of such studies provides data for the government and nongovernment sectors to recognize proprieties and consider strategies, combining precise surveillance and limitations of these zoonotic infections.

## Figures and Tables

**Table 1:** Logistic regression analysis of risk factors associated with toxocariasis among general population from Abadan and Khorramshahr cities (Khuzestan Province, southwest Iran).

Variable	No. of tested (%)	Seroprevalence		Univariate analysis			Multivariate analysis		
		%	95% CI	COR	95% CI	*p* value ^*∗*^	AOR	95% CI	*p* value ^*∗*^
Age group (years)									
<20	45 (12.4%)	13 (28.9%)	17.73–43.37	2.55	0.92–7.12	0.073	3.69	0.84–16.29	0.084
20–39	157 (43.3%)	11 (7.0%)	3.96–12.11	0.47	0.17–1.30	0.145	0.59	0.17–2.03	0.404
40–59	110 (30.3%)	15 (13.6%)	8.44–21.29	0.99	0.38–2.61	0.988	1.08	0.34–3.45	0.898
≥60	51 (14.0%)	7 (13.7%)	6.81–25.72	Ref	—	—	Ref	—	—
Gender									
Male	173 (47.7%)	26 (15.0%)	10.47–21.11	1.49	0.81–2.78	0.20	1.47	0.70–3.03	0.314
Female	190 (52.3%)	20 (10.5%)	6.92–15.70	0.67	0.36–1.24	—	0.68	0.33–1.43	—
Education level									
Diploma or lower	260 (71.6%)	37 (14.2%)	10.50–19.0	1.72	0.81–3.73	0.160	0.34	0.11–1.05	0.061
University degree	103 (28.4%)	9 (8.7%)	4.66–15.78	0.58	0.27–1.24	—	2.91	0.95–8.92	—
Residence area									
Urban	292 (80.4%)	22 (7.5%)	5.03–11.14	0.16	0.08–0.31	<0.001	0.54	0.15–1.89	0.336
Rural	71 (19.6%)	24 (33.8%)	23.88–45.38	6.27	3.25–12.08	—	1.86	0.53–6.57	—
Source of drinking water									
Purified water	309 (85.1%)	27 (8.7%)	6.07–12.41	0.18	0.09–0.35	<0.001	0.47	0.15–1.47	0.192
Unpurified water	54 (14.9%)	19 (35.2%)	23.82–48.51	5.67	2.86–11.23	—	2.15	0.68–6.81	—
Contact with dog									
Yes	252 (69.4%)	45 (17.9%)	13.62–23.06	23.91	3.23–100.0	0.002	3.85	0.39–33.33	0.246
No	111 (30.6%)	1 (0.9%)	0.2–4.93	0.04	0.01–0.31	—	0.26	0.03–2.54	—
Contact with soil									
Yes	127 (35.0%)	37 (29.1%)	21.94–37.56	10.0	4.76–25.0	<0.001	2.94	0.99–9.09	0.053
No	236 (65.0%)	9 (3.8%)	2.02–7.09	0.10	0.04–0.21	—	0.34	0.11–1.01	—
Eating improperly washed vegetables									
Yes	149 (41.0%)	36 (24.2%)	17.99–31.63	6.67	3.12–14.29	<0.001	1.33	0.34–5.26	0.685
No	214 (59.0%)	10 (4.7%)	2.56–8.39	0.15	0.07–0.32	—	0.75	0.19–2.98	—
History of drinking untreated water									
Yes	156 (43.0%)	37 (23.7%)	17.72–30.97	6.67	3.23–14.29	<0.001	1.3	0.34–5.0	0.698
No	207 (57.0%)	9 (4.3%)	2.30–8.05	0.15	0.07–0.31	—	0.77	0.20–2.95	—
Having information about toxocariasis and its transmission routes									
Yes	22 (6.1%)	2 (9.1%)	2.53–27.81	0.68	0.15–3.03	0.605	0.44	0.08–2.33	0.335
No	341 (93.9%)	44 (12.9%)	9.75–16.88	1.48	0.33–6.56	—	2.27	0.43–12.04	—

COR, crude odds ratio; AOR, adjusted odds ratio; CI, confidence interval.  ^*∗*^*p*-value < 0.05 indicates a significant difference in seroprevalence between the categories within each characteristic.

**Table 2:** Clinical symptoms associated with toxocariasis in general population from Abadan and Khorramshahr cities (Khuzestan Province, southwest Iran).

Variable	No. of tested (%)	Seroprevalence		OR (95% CI)	*p* value
		%	95% CI		
Asthma					
Yes	93 (25.6%)	27 (29.0%)	20.78–38.94	5.26 (2.86–10.0)	
No	270 (74.4%)	19 (7.0%)	4.55–10.73	0.19 (0.10–0.35)	<0.001
Skin allergic disorders					
Yes	102 (28.1%)	30 (29.4%)	21.44–38.87	6.25 (3.33–12.50)	
No	261 (71.9%)	16 (6.1%)	3.81–9.72	0.16 (0.08–0.30)	<0.001
Ophthalmic disorders or blurred vision					
Yes	77 (21.2%)	34 (44.2%)	33.60–55.26	16.67 (9.09–33.33)	
No	286 (78.8%)	12 (4.2%)	2.42–7.19	0.06 (0.03–0.11)	<0.001
Epilepsy					
Yes	15 (4.1%)	3 (20.0%)	7.05–45.18	1.79 (0.48–6.67)	
No	348 (95.9%)	43 (12.4%)	9.30–16.23	0.56 (0.15–2.08)	0.390

OR, odds ratio; CI, confidence interval.

## Data Availability

The data used to support the findings of this study are available from the corresponding author upon reasonable request.

## Additional Points

*Limitations*. In this study, the only ELISA assay was performed on collected sera with no supporting data by other confirmatory techniques.
